# Quality of clinical guidelines: It matters as it impacts patient care

**DOI:** 10.1002/ueg2.12606

**Published:** 2024-06-04

**Authors:** Iris J. M. Levink, Alberto Balduzzi, Irene Marafini, Haluk Tarik Kani, Yasuko Maeda, UEGJ Guideline Taskforce

**Affiliations:** ^1^ Department of Gastroenterology and Hepatology Erasmus MC University Medical Centre Rotterdam The Netherlands; ^2^ Department of Internal Medicine Reinier de Graaf Gasthuis Delft The Netherlands; ^3^ Department of Surgery, Dentistry, Paediatrics and Gynaecology Unit of General and Pancreatic Surgery The Pancreas Institute Verona University of Verona Verona Italy; ^4^ Policlinico Universitario Tor Vergata Gastroenterology Unit Rome Italy; ^5^ Department of Gastroenterology Marmara University School of Medicine Istanbul Turkey; ^6^ Marmara University Institute of Gastroenterology Istanbul Turkey; ^7^ Department of Surgery Queen Elizabeth University Hospital Glasgow UK

**Keywords:** AGREE II, assessment, gastroenterology, GRADE, guideline, quality

Clinical guidelines are of vital importance in providing healthcare professionals with updated evidence‐based recommendations eventually aiming to improve patient care and outcomes. Nonetheless, guidelines often lack evidence or transparency in how evidence was interpreted and graded. Historically, the development of guidelines was done in GOBSAT ‐ (‘Good old men sitting around the table’) style by collating data from literature mixed with experience‐based opinions. These guidelines often fail to address some of the essential and pivotal factors that are fundamental to high‐quality guidelines. In addition, they often lack applicability, practicality, and the outcomes that really matter to patients.

In recent decades, there has been a shift to making the guideline development process more transparent, robust, and evidence‐based, whilst involving multiple stakeholders (e.g., patients, general practitioners, or paramedics). Within the editorial board of United European Gastroenterology (UEG) Journal, a Guidelines Taskforce was established that aims at fostering a heightened awareness among readership and contributing authors on the quality of guidelines and the critical importance of transparency throughout the guideline development process.

In order to scrutinize the adherence to the standards of guidelines within the field of gastroenterology, the Taskforce collected and evaluated all 16 guidelines^1^, ^2^, ^3^, ^4^, ^5^, ^6^, ^7^, ^8^, ^9^, ^10^, ^11^, ^12^, ^13^, ^14^, ^15^, ^16^, ^17^ published in the UEG Journal between January 2020 and March 2022. The assessment was conducted by four reviewers, who were equipped with the necessary expertise through participation in the International Guideline Training and Certification (INGUIDE) program (https://inguide.org/).

The four reviewers used the Appraisal of Guidelines for Research Evaluation II (AGREE II) tool,^18^ which is a widely acknowledged instrument that provides both authors and (external) reviewers with a standardized methodology for assessing the critical quality metrics of guidelines. It features a detailed checklist designed to improve the reporting of guidelines, structured around six key domains:

1. Scope and purpose
2. Stakeholder involvement
3. Rigor of development
4. Clarity of presentation
5. Applicability
6. Editorial independence

These domains collectively address the essential components that underpin the reliability and validity of guidelines. The scoring is performed using a 7‐point scale (ranging from 1: Strongly Disagree to 7: Strongly Agree) based on specific items within the domain itself. Each domain is scored by summing up all the scores of the items within the domain and scaling the total as a percentage of the maximum possible score. This results in one score per guideline per domain.

Overall, the reviewed guidelines were of good quality, mostly achieving above 70% in each domain (Figure 1). The highest scores were reached in **Domain 1—scope and purpose** (median 80%), as the scope and purpose of each guideline was generally explicit. Strikingly, the objectives of guidelines were not always specific to the clinical topic described in the guideline. For instance, a more specific objective could be ‘to lower the risk of involuntary loss of stool’ rather than ‘to improve patient outcomes’.

**FIGURE 1:**
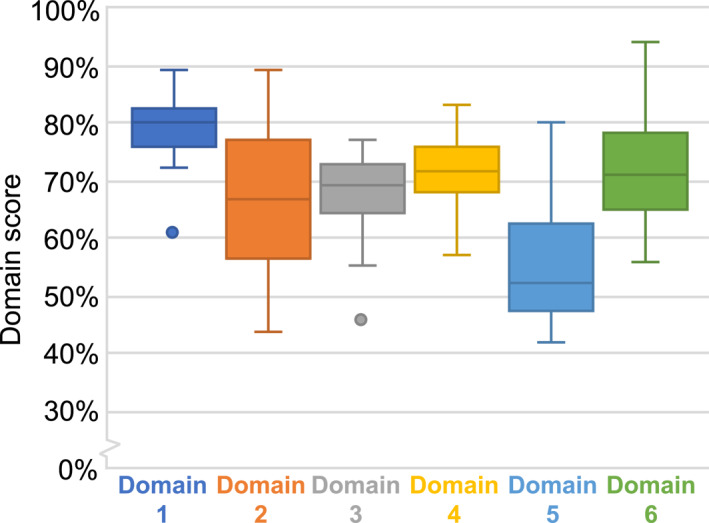
Boxplot showing median and ranges of AGREE II scores per domain for the 16 included guidelines.

The lowest scores were observed in **Domain 5—applicability** (median 53%). The domain includes factors, such as, whether the guideline provides recommendations or a tool on how to implement guidelines in practice, how to overcome facilitators/barriers and their cost implications. Most of the assessed guidelines lacked evaluation of barriers leading to reduced applicability of the guidelines. Performing pilot testing of the guideline or seeking feedback from key stakeholders or a health economist may highlight challenges in applicability and facilitate early adjustments before widespread implementation.

**Domain 3—rigor of development** (median 70%) is often considered as the most critical domain. Multiple frameworks are being used to appraise the quality of available evidence. To be relevant and practical, guidelines must follow a solid framework, involving a transparent systematic review process and a clear presentation of the evidence strength and recommendation rationale. The most frequently used framework is Grading of Recommendations Assessment, Development and Evaluation (GRADE).^19^ It provides a systematic approach for developing and presenting evidence, has gained widespread acceptance and is currently endorsed by over 100 organizations, including the World Health Organization and the National Institute for Health and Care Excellence. Most of the reviewed guidelines indeed attempted to use GRADE, but did not apply it thoroughly. There is a scope to promote further training and education, such as INGUIDE (https://inguide.org/) or the UEG guideline development course (https://ueg.eu/p/269), prior to taking part in guideline development.

To further increase transparency, we would recommend the use of an Evidence‐to‐Decision framework, which provides the reader background, research evidence, considerations from the panel members, and drawn conclusions. To ensure the methodological rigor, (preferably multiple) external experts with different expertise (including a methodologist) could be involved.

Overall, guidelines generally had a multidisciplinary approach involving multiple stakeholders (covered in **Domain 2—stakeholder involvement**; median 67%), yet roles of the stakeholders within the guideline panel were frequently not clear, as these were not always explicitly stated in the published articles. The most important health questions can be missed if representatives of one or more disciplines are missing.

**Domain 4—clarity of presentation** (median 72%) and **Domain 6—editorial independence** (median 71%) both had medium to high scores. **Domain 4** appraises the quality of recommendations, which should be specific and unambiguous. Guidelines often lacked specification of the intent or purpose of a recommendation (e.g., ‘to improve quality of life’).

In conclusion, this editorial underscores the importance of standardized guideline development in order to improve transparency, rigor and applicability. The Quality of Care Committee of UEG is actively promoting educational events (https://ueg.eu/p/269), tools and frameworks to facilitate this process,^20^ ultimately aiming at a more uniform landscape of high‐quality clinical guidance.

## CONFLICT OF INTEREST STATEMENT

Speaking fee from Galapagos and Abbvie. Haluk Tarik Kani received speaking fee from Janssen and Abbvie.

## Data Availability

The data that support the findings of this study are available on request from the corresponding author. The data are not publicly available due to privacy or ethical restrictions.
